# Parity and Number of Teeth in Japanese Women: Results from the Japan Nurses' Health Study

**DOI:** 10.1089/whr.2020.0066

**Published:** 2020-09-15

**Authors:** Akira Taguchi, Kazue Nagai, Yuki Ideno, Takumi Kurabayashi, Kunihiko Hayashi

**Affiliations:** ^1^ Department of Oral and Maxillofacial Radiology, School of Dentistry, Matsumoto Dental University, Shiojiri, Japan.; ^2^ Department of Hard Tissue Research, Graduate School of Oral Medicine, Matsumoto Dental University, Shiojiri, Japan.; ^3^ School of Health Sciences, Gunma University, Maebashi, Japan.; ^4^ Gunma University Center for Mathematics and Data Science, Maebashi, Japan.; ^5^ Department of Obstetrics and Gynecology, Niigata City General Hospital, Niigata, Japan.

**Keywords:** nurse, parity, risk factor, tooth, women

## Abstract

***Background:*** Parity is thought to be associated with a decreased number of teeth present in women. However, educational level and social status, which are particularly significant risk factors for loss of teeth, have been heterogeneous in previous observations. This cross-sectional survey aimed to clarify the associations of parity with the risk of having <20 teeth in Japanese female nurses participating in the Japan Nurses' Health Study (JNHS).

***Methods:*** In the third follow-up questionnaire of the JNHS, 11,299 women aged 27–82 years participated in this study. The number of participants according to age range was 7,225 (63.9%) aged <50 years and 4,074 (36.1%) aged ≥50 years. Information on parity and risk factors for loss of teeth was collected through a baseline questionnaire and then a follow-up questionnaire. Multivariate logistic regression analysis was used to calculate the adjusted odds ratio (OR) and 95% confidence interval (CI) of having <20 teeth according to parity category.

***Results:*** Participants ≥50 years who had experienced three or more deliveries had a significantly higher risk of having <20 teeth than those who had not experienced delivery (OR = 1.59, 95% CI = 1.14–2.20), although this finding was not observed in participants <50 years. In addition to parity, age and current smoking may be independent risk factors for having <20 teeth in Japanese nurses.

***Conclusions:*** Higher-parity female nurses ≥50 years may be more likely to lose teeth than those who have not experienced delivery.

## Introduction

Loss of teeth impairs occlusal function. Japanese people >80 years of age with at least 20 teeth generally have relatively good occlusion.^1^ In addition, the presence of 20 or more teeth beneficially affects various functions other than mastication.^2–4^ Older adults with ≥20 teeth have a lower risk of onset of functional disability, a lower risk of incident falls, and better cognitive function. Maintaining 20 or more teeth is an important factor in protecting a range of functions. A political initiative encouraging the preservation of 20 or more teeth at the age of 80 years has been introduced by the Ministry of Health, Labour and Welfare of Japan.^5^

Poor oral health habits, such as low frequency of tooth brushing, no routine dental checkups, current smoking, and low educational status are independent risk factors for having <20 teeth in the general population of individuals aged 40 years and older in Japan.^6^ Lower daily frequency of tooth brushing and current smoking were associated with a decreased number of teeth present in 3,767 Korean adults aged 55–84 years.^7^ In addition, factors associated with hormonal changes during pregnancy and after menopause in women affect the immune response to bacterial plaque and induce vascular and gingival changes that may contribute to heightened gingival inflammation, which results in periodontal disease progression and loss of teeth.^8^ Meisel et al. reported that the bone turnover rate was positively related to the number of children born in the youngest age group (20–40 years).^9^ In postmenopausal women treated with estrogens, the number of teeth was higher than in men of the same age group. They concluded that the apparent paradox of having fewer teeth despite better periodontal health in women compared with men may be related to an increased bone turnover rate and socioeconomic conditions such as low education and low social status.

Some investigators have reported an association of parity and pregnancy with the number of teeth remaining in women.^10–12^ In previous reports, educational level and social status, both particularly significant risk factors for loss of teeth, were adjusted by use of statistical procedures; however, it is unclear whether sufficient statistical adjustment was conducted. It is ideal for such studies to be performed in a group with educational level and social status that are as uniform as possible. We, therefore, investigated the association between parity and the risk of having <20 teeth present in a large number of Japanese female nurses participating in the Japan Nurses' Health Study (JNHS). Because the decrease in estrogens after menopause may contribute to the progression of periodontal disease,^9^ it is likely that the combination of menopause with parity may increase the risk of loss of teeth more than parity alone. Because the mean menopausal age in the JNHS is 50 years,^13^ women who participated in this study were divided into two groups: <50 years and ≥50 years.

## Methods

### Study protocol and participants

The JNHS is a large prospective cohort study designed to investigate the effects of lifestyle and health care practices on the health of Japanese women.^14^ Participants were recruited from 2001 through 2007. A total of 49,927 women from all 47 prefectures in Japan responded to the baseline survey. Among them, 15,019 women agreed to submit to follow-up and returned signed informed consent sheets, together with their completed baseline questionnaires. The study population comprised women at least 25 years old who practiced as registered nurses, licensed practical nurses, public health nurses, and/or midwives, and who resided in Japan at the time of the baseline survey. The JNHS coordination and data center is located at the Epidemiological Research Office of the School of Health Sciences at Gunma University, Japan. The JNHS study was approved by the Ethics Committee of Gunma University. The methods were carried out in accordance with the relevant guidelines. All procedures performed in studies involving human participants were in accordance with the ethical standards of the institutional and/or national research committee and with the 1964 Helsinki declaration and its later amendments or comparable ethical standards. Informed consent was obtained from all individual participants included in the study.

**FIG. 1.**
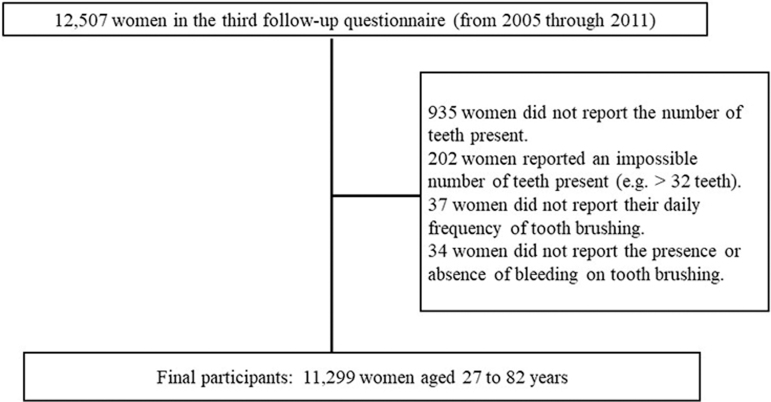
Participants registration chart showing the number of participants in this clinical study.

In the third follow-up questionnaire (from 2005 to 2011), we asked 12,507 participants to report their number of teeth present and frequency of tooth brushing per day. We previously demonstrated a high correlation (*r* = 0.82) between the actual and self-reported number of teeth present in 200 Japanese people aged 50–90 years.^15^ Out of the 12,507 women, 935 women did not report their number of teeth present and 202 women reported an impossibly incorrect number of teeth present (>32 teeth). Thirty-seven women did not report their daily frequency of tooth brushing and 34 did not report the presence or absence of bleeding on tooth brushing. Twenty-nine edentulous women were included in the 37 women who did not report their daily frequency of tooth brushing. Finally, 11,299 women aged 27–82 years participated in this study (Fig. 1). Information on parity was collected at baseline. Information on potential risk factors for loss of teeth, such as age, body mass index (BMI), current smoking status, daily frequency of tooth brushing, bleeding on tooth brushing, presence of diabetes mellitus, and final educational status, was collected through a follow-up self-administered questionnaire. Information about marital status and workplace was also collected to evaluate the participants' social status.

### Statistical analysis

Continuous variables are expressed as means ± standard deviation (SD). The chi-squared test, *t*-test, and Wilcoxon rank sum test were used to investigate differences in age, age category (40–49, 50–59, 60–69, and 70–79 years), number of teeth present, BMI (kg/m^2^), parity (0, 1, 2, 3, or more deliveries, or missing data), current smoking (yes, no, or missing data), daily frequency of tooth brushing (1, 2, and 3 or more times), bleeding on tooth brushing (yes or no), diabetes mellitus (yes or no), and final educational status (high school, vocational college, junior college, university, or missing data) between participants who had ≥20 teeth and <20 teeth. The chi-squared test, *t*-test, and Wilcoxon rank sum test were also used to investigate differences in factors related to oral health, including age, BMI (kg/m^2^), current smoking (yes, no, or missing data), daily frequency of tooth brushing (1, 2, or 3 or more times), diabetes mellitus (yes or no), and final educational status (high school, vocational college, junior college, university, or missing data), among participants who had had 0, 1, 2, 3, or more deliveries, or were missing delivery data.

Participants who had ≥20 teeth were compared with those who had <20 teeth in this study. In addition, participants were divided into two groups: <50 years and ≥50 years. Multivariate logistic regression analysis, adjusted for age, BMI (kg/m^2^), current smoking (three categories), daily frequency of tooth brushing (three categories), bleeding on tooth brushing (binary), diabetes mellitus (binary), and final educational status (five categories), was used to calculate the adjusted odds ratio (OR) and 95% confidence interval (CI) of having <20 teeth according to the parity category. To test the robustness of our findings, we performed sensitivity analysis by using a multivariate linear regression model in which the number of teeth present was an independent continuous variable. All comparisons were two sided and performed at a *p* = 0.05 level of significance. Statistical analysis was performed using SAS version 9.4 (SAS Institute, Cary, NC).

## Results

### Characteristics of study participants

The characteristics of the 11,299 study participants are shown in Table 1. Mean age (SD) was 46.4 (8.2) years and mean number of teeth present (SD) was 26.3 (4.6). The number of participants according to age range was 2,654 (23.5%) aged <40 years, 4,571 (40.5%) aged between 40 and 49 years, and 3,405 (30.1%) aged between 50 and 59 years. Only 669 participants (6%) were aged between 60 and 82 years. The number of participants according to parity was 3,100 (27.4%) for no delivery, 1,425 (12.6%) for one delivery, 3,969 (35.1%) for two, and 2,530 (22.4%) for three or more deliveries. Of all participants, 1,279 participants (11.3%) were currently smoking, 4,680 (41.4%) experienced bleeding on tooth brushing, and 7,210 (63.8%) brushed their teeth three or more times a day. Based on BMI, the number of underweight and overweight participants was 882 (7.8%) and 1,683 (14.9%), respectively. The number of high school graduates, vocational college graduates, junior college graduates, and university graduates was 29 (0.3%), 8,685 (76.9%), 1,729 (15.3%), and 463 (4.1%), respectively. One hundred and thirty-one participants (1.2%) had diabetes mellitus.

**Table 1.** Characteristics of 11,299 Study Participants

	Results
Age (years)	46.4 ± 8.2
No. of teeth present	26.3 ± 4.6
Age category, years	
<40	2654 (23.5)
40–49	4571 (40.5)
50–59	3405 (30.1)
60–69	640 (5.7)
>69	29 (0.3)
No. of deliveries	
Missing	275 (2.4)
0	3100 (27.4)
1	1425 (12.6)
2	3969 (35.1)
≥3	2530 (22.4)
Current smoking	
Missing	10 (0.1)
No	10010 (88.6)
Yes	1279 (11.3)
Bleeding on tooth brushing	
No	6619 (58.6)
Yes	4680 (41.4)
Daily frequency of tooth brushing	
1	452 (4.0)
2	3637 (32.2)
≥3	7210 (63.8)
BMI (kg/m^2^)	
Missing	80 (0.7)
<18.5	882 (7.8)
18.5–24.9	8654 (76.6)
≥25.0	1683 (14.9)
Final education status	
Missing	393 (3.5)
High school	29 (0.3)
Vocational college	8685 (76.9)
Junior college	1729 (15.3)
University	463 (4.1)
Diabetes mellitus	
Missing	5 (0.0)
Yes	131 (1.2)
No	11163 (98.8)
Marital status	
Missing	110 (1.0)
Unmarried	2216 (19.6)
Married	8011 (70.9)
Divorced	723 (6.4)
Separated	58 (0.5)
Bereaved	186 (1.6)
Workplace	
Missing	79 (0.7)
Hospital	9596 (84.9)
Clinic	164 (1.5)
Educational institution	366 (3.2)
Company/office	103 (0.9)
Local administration	607 (5.4)
Public health and welfare office	62 (0.5)
Other	322 (2.9)

Results are given as the mean ± SD or number of participants (%).

BMI, body mass index; SD, standard deviation.

The number of married and unmarried participants was 8,011 (70.9%) and 2,216 (19.6%), respectively. Other categories included divorced, separated, and bereaved. One hundred and ten participants (1.0%) had missing data. Regarding the workplace, 9,596 participants (84.9%) worked in a hospital, 164 participants (1.5%) in a clinic, 366 participants (3.2%) in an educational institution, 103 participants (0.9%) in a company/office, 607 participants (5.4%) in local administration, 62 participants (0.5%) in the public health and welfare office, and 322 participants (2.9%) in other locations. Seventy-nine participants (0.7%) had missing data.

Participants who had <20 teeth were significantly older than those who had ≥20 teeth (51.9 ± 8.0 vs. 46.0 ± 8.1, *p* < 0.001) (Table 2). In addition, participants with <20 teeth had a significantly higher frequency of delivery (*p* < 0.001), rate of current smoking (*p* < 0.001), frequency of bleeding on tooth brushing (*p* < 0.001), frequency of being overweight (*p* < 0.001), and frequency of high school graduation (*p* = 0.005) than those with 20 or more teeth. There was no significant difference in the presence of diabetes mellitus between participants who had <20 teeth and 20 or more teeth.

**Table 2.** Differences in Some Factors Between Participants with <20 Teeth and Those with ≥20 Teeth

	<20 Teeth, N = 786	≥20 Teeth, N = 10,513	p
Age (years)	51.9 ± 8.0	46.0 ± 8.1	<0.001
No. of teeth present	13.5 ± 4.7	27.2 ± 2.9	<0.001
Age category, years			
<40	56 (7.1)	2598 (24.7)	<0.001
40–49	222 (28.2)	4349 (41.4)	
50–59	387 (49.2)	3018 (28.7)	
60–69	111 (14.1)	529 (5.0)	
>69	10 (1.3)	19 (0.2)	
No. of deliveries			
Missing	21 (2.7)	254 (2.4)	<0.001
0	127 (16.2)	2973 (28.3)	
1	90 (11.5)	1335 (12.7)	
2	316 (40.2)	3653 (34.8)	
≥3	232 (29.5)	2298 (21.9)	
Current smoking			
Missing	1 (0.1)	9 (0.1)	<0.001
No	649 (82.6)	9361 (89.0)	
Yes	136 (17.3)	1143 (10.9)	
Bleeding on tooth brushing			
No	405 (51.5)	6214 (59.1)	<0.001
Yes	381 (48.5)	4299 (40.9)	
Daily frequency of tooth brushing			
1	37 (4.7)	415 (4.0)	0.131
2	266 (33.8)	3371 (32.1)	
≥3	483 (61.5)	6727 (64.0)	
BMI (kg/m^2^)			
Missing	5 (0.6)	75 (0.7)	<0.001
<18.5	50 (6.4)	832 (7.9)	
18.5–24.9	573 (72.9)	8081 (76.9)	
≥25.0	158 (20.1)	1525 (14.5)	
Final education status			
Missing	34 (4.3)	359 (3.4)	0.005
High school	9 (1.2)	20 (0.2)	
Vocational college	613 (78.0)	8072 (76.8)	
Junior college	100 (12.7)	1629 (15.5)	
University	30 (3.8)	433 (4.1)	
Diabetes mellitus			
Missing	1 (0.1)	4 (0.0)	0.124
Yes	14 (1.8)	117 (1.1)	
No	771 (98.1)	10392 (98.9)	

Results are given as the mean ± SD or number of participants (%).

### Association of parity with oral health factors and of parity and other factors with the risk of having <20 teeth

Significant differences were observed in age (*p* < 0.001), BMI (*p* < 0.001), current smoking (*p* < 0.001), final educational status (*p* < 0.001), and presence of diabetes mellitus (*p* < 0.001), but not in daily frequency of tooth brushing (*p* = 0.30) among participants who had 0, 1, 2, ≥3 deliveries, or were missing delivery data (Table 3).

**Table 3.** Differences in Age, Current Smoking Habit, Daily Frequency of Tooth Brushing, Body Mass Index, Final Educational Status, and Diabetes Mellitus Among Participants Who Had 0, 1, 2, ≥3 Deliveries, and Missing

	No. of deliveries					p
	Missing	0	1	2	≥3	
Age (years)	45.2 ± 9.7	41.8 ± 7.8	44.9 ± 8.1	49.8 ± 7.5	49.4 ± 6.5	<0.001
Current smoking						
Missing	0	0	0	0.2	0	<0.001
No	85.1	82.1	87.7	88.9	90.8	
Yes	14.9	12.9	12.4	10.9	9.1	
Daily frequency of tooth brushing						
1	6.9	3.6	4.7	4.1	3.6	0.300
2	29.8	32.8	33.4	30.9	33.0	
≥3	63.3	63.7	61.9	65.0	63.4	
BMI (kg/m^2^)						
Missing	1.1	0.7	0.8	0.7	0.7	<0.001
<18.5	13.1	11.5	9.1	6.4	4.3	
18.5–24.9	70.2	74.4	75.6	78.5	77.6	
≥25.0	15.6	13.4	14.6	14.5	17.5	
Final education status						
Missing	2.9	1.8	3.0	4.5	4.3	<0.001
High school	0.7	0.0	0.1	0.4	0.4	
Vocational college	80.4	76.5	76.6	77.3	76.4	
Junior college	12.4	15.5	16.3	14.6	16.0	
University	3.6	6.3	3.9	3.2	3.0	
DM						
Missing	0.7	0.0	0.0	0.0	0.1	<0.001
Yes	2.2	0.8	0.6	1.4	1.4	
No	97.1	99.2	99.4	98.6	98.5	

Results are given as the mean ± SD or % participants.

DM, diabetes mellitus.

Age was significantly associated with an increased risk of having <20 teeth in participants <50 years and participants ≥50 years (Tables 4 and 5). There was no significant association of parity with the risk of having <20 teeth in participants <50 years (Table 4). Participants ≥50 years who had experienced three or more deliveries had a significantly higher risk of having <20 teeth than those ≥50 years who had never delivered (OR = 1.59; 95% CI = 1.14–2.20) (Table 5). A current smoking habit and bleeding on tooth brushing were significantly associated with an increased risk of having <20 teeth in participants aged <50 years and participants ≥50 years. There was no significant association between the risk of having <20 teeth and BMI in participants <50 years. Overweight participants (BMI ≥25) ≥50 years had a significantly higher risk of having <20 teeth than those with normal BMI (18.5–24.9). There was no significant association between the risk of having <20 teeth and daily frequency of tooth brushing in participants <50 years and ≥50 years. High school graduates had an increased risk of having <20 teeth compared with university graduates, although only 27 high school graduates participated in the study. The presence of diabetes mellitus was not significantly associated with an increased risk of having <20 teeth in participants <50 years and participants ≥50 years. Sensitivity analysis showed the robustness of our findings in this study.

**Table 4.** Crude and Adjusted Odds Ratios for Having <20 Teeth Using Multivariate Logistic Regression Analysis in 7,225 Participants <50 Years

	Crude OR			Adjusted OR		
	OR	95% CI lower	95% CI upper	OR	95% CI lower	95% CI upper
Age (years)	1.10	1.07	1.13	1.09	1.06	1.12
Delivery						
Missing	1.39	0.63	3.07	1.30	0.59	2.90
0	Ref.			Ref.		
1	1.55	1.06	2.28	1.36	0.92	2.01
2	1.73	1.27	2.36	1.27	0.91	1.76
≥3	1.58	1.11	2.26	1.06	0.72	1.55
Current smoking						
Missing^a^						
No	Ref.			Ref.		
Yes	2.39	1.80	3.17	2.31	1.73	3.08
Bleeding on brushing						
No	Ref.			Ref.		
Yes	1.39	1.10	1.77	1.40	1.10	1.79
Daily frequency of brushing						
1	1.01	0.53	1.94	0.84	0.43	1.61
2	1.03	0.80	1.33	0.97	0.75	1.27
≥3	Ref.			Ref.		
BMI						
Missing	0.43	0.06	3.17	0.56	0.08	4.06
<18.5	0.71	0.44	1.14	0.80	0.50	1.30
18.5–24.9	Ref.			Ref.		
≥25.0	1.23	0.89	1.71	1.14	0.81	1.59
Final education status						
Missing	1.41	0.60	3.34	0.98	0.41	2.35
High school^a^						
Vocational college	1.02	0.57	1.86	0.96	0.53	1.75
Junior college	0.80	0.41	1.55	0.82	0.42	1.60
University	Ref.			Ref.		
DM						
Missing^a^						
Yes	0.86	0.12	6.34	0.76	0.10	5.72
No	Ref.			Ref.		

^a^ There were no participants with <20 teeth in the missing data for current smoking, two participants with <20 teeth in the high school graduation for final education status, and one participant with <20 teeth in the missing data for DM. We cannot calculate ORs and CIs in these categories because of small number of participants.

CI, confidence interval; OR, odds ratio; Ref., reference.

**Table 5.** Crude and Adjusted Odds Ratios for Having <20 Teeth Using Multivariate Logistic Regression Analysis in 4,074 Participants ≥50 Years

	Crude OR			Adjusted OR		
	OR	95% CI lower	95% CI upper	OR	95% CI lower	95% CI upper
Age (years)	1.07	1.05	1.09	1.08	1.06	1.11
Delivery						
Missing	1.54	0.82	2.91	1.25	0.65	2.42
0	Ref.			Ref.		
1	1.15	0.76	1.74	1.11	0.73	1.69
2	1.13	0.83	1.54	1.17	0.85	1.61
≥3	1.42	1.03	1.95	1.59	1.14	2.20
Current smoking						
Missing	7.42	0.46	118.81	8.18	0.50	133.00
No	Ref.			Ref.		
Yes	1.65	1.24	2.19	1.88	1.40	2.52
Bleeding on brushing						
No	Ref.			Ref.		
Yes	1.54	1.28	1.86	1.66	1.37	2.01
Daily frequency of brushing						
1	1.23	0.80	1.87	0.98	0.63	1.52
2	1.18	0.97	1.45	1.00	0.82	1.23
≥3	Ref.			Ref.		
BMI						
Missing	1.71	0.56	5.09	2.01	0.67	6.06
<18.5	1.48	0.99	2.21	1.46	0.96	2.21
18.5–24.9	Ref.			Ref.		
≥25.0	1.42	1.13	1.78	1.35	1.07	1.71
Final education status						
Missing	0.98	0.51	1.87	0.88	0.45	1.70
High school	3.72	1.46	9.52	2.86	1.09	7.54
Vocational college	1.07	0.65	1.78	1.04	0.62	1.73
Junior college	0.95	0.54	1.65	0.93	0.53	1.63
University	Ref.			Ref.		
DM						
Missing	2.35	0.24	22.59	1.99	0.20	19.96
Yes	1.04	0.58	1.88	0.79	0.43	1.45
No	Ref.			Ref.		

## Discussion

Our findings showed that Japanese female nurses ≥50 years who had experienced three or more deliveries had a higher risk of having <20 teeth than those ≥50 years who had never delivered. This result is in accordance with a previous Japan public health center-based oral health study of 649 women ≥55 years that reported that women with four or more children lose nearly three more teeth than women with no children or one child.^11^ The results of both studies suggest that higher-parity women ≥50 years are more likely to lose teeth than lower-parity women ≥50 years. However, there was no significant association of parity with the risk of having <20 teeth in female nurses <50 years in our study. This implies that a decrease of estrogen may exert an additive effect for the association between parity and the risk of having <20 teeth.

Morelli et al. described several possible explanations for an association between higher parity and tooth loss in women.^8^ Pregnancy is associated with rising noncyclic levels of sex hormones. Hormonal changes during pregnancy affect the immune response to bacterial plaque. This results in vascular and gingival changes that may contribute to heightened gingival inflammation. It is possible that repeated gingival inflammation leading to periodontal disease may be associated with an increased risk of tooth loss. In fact, the presence of bleeding on tooth brushing, indicating the presence of periodontal disease, significantly increased the risk of having <20 teeth in our current study.

In addition to periodontal disease, dental caries is a major risk factor for loss of teeth. Ueno et al. found no significant association between parity and total dental caries, including decayed and filled teeth.^11^ Russell et al. conducted a study of 2,635 women selected from the NHANES III data set and found that although increased parity was not associated with a greater level of total dental caries, parity was related to untreated dental caries.^16^ Untreated dental caries associated with parity may result in a decreased number of teeth present.

Another potential mechanism for tooth loss is the participants' education level, which is an important determining factor for women's fertility rate, with a gradient associating fewer children with higher educational attainment.^8^ High school graduates had an increased risk of having <20 teeth in comparison with university graduates in participants ≥50 years in our study. Ishikawa et al.^6^ divided participants into two educational groups: the high educational group (any college or higher educational graduation) and the low educational group (up to high school graduation). They concluded that low educational status was a particularly significant risk factor for <20 teeth.^6^ Their result is in accordance with our result, although the number of high school graduates in our study was too small (0.3%) to compare. In the study of Ishikawa et al., the number of participants in the high educational group was smaller than that in the low educational group (1,500 vs. 6,042).^6^ According to their educational group definition, most of our participants (96.3%) belonged to the high educational group. The significant association of higher parity with increased risk of having <20 teeth in participants ≥50 years was observed even in this redefined high educational group.

Smoking is an important determinant for the loss of teeth in both men and women.^17–19^ A current smoking habit was significantly associated with having <20 teeth in 7,542 community-dwelling Japanese people aged 40 years and older (adjusted OR = 1.68, 95% CI = 1.41–1.99) in the Yamagata study.^6^ Our results are consistent with those of the Yamagata study. Parity was significantly associated with the decreasing rate of current smoking in our study. Women who have had three or more deliveries may be more likely to give up smoking than those who have never delivered, although women with three or more deliveries aged ≥50 years had a significantly increased risk of having <20 teeth than women who had never delivered aged ≥50 years in our study.

We found no significant association between the risk of having <20 teeth and daily frequency of tooth brushing, although the risk of having <20 teeth was significantly associated with daily frequency of tooth brushing in the Yamagata study.^6^ Approximately 64% of women in our study brushed their teeth three or more times a day compared with only 13% in the Yamagata study, which implies that most women in our study had good oral health habits that are likely to result in the retention of teeth. Parity was not significantly associated with daily frequency of tooth brushing in our study. Parity among female nurses may not influence attitudes to oral hygiene management.

In this study, overweight women ≥50 years had a significantly higher risk of having <20 teeth than nonoverweight women ≥50 years. In our previous study, overweight postmenopausal women had an increased risk of tooth loss, despite their higher skeletal bone mineral density, when compared with women with normal BMI.^20^ Recent meta-analysis demonstrated that this positive association was consistent and coherent with a biologically plausible role for obesity in the development of periodontal disease.^21^ Parity was significantly associated with increasing BMI in our study. Progression of periodontal disease caused by overweight or obesity may contribute to the loss of teeth in women.

It is well known that diabetes mellitus aggravates periodontal disease, which results in an increased risk of tooth loss.^22,23^ Suzuki et al. recently performed a population-based survey using information obtained from the National Database of Health Insurance Claims and Specific Health Checkups in Japan and concluded that patients in the diabetes mellitus group had a higher level of tooth loss than patients in the control group, among both genders.^24^ However, Ishikawa et al. found no significant association between diabetes mellitus (OR = 1.06, 95% CI = 0.83–1.35, *p* = 0.65) and having <20 teeth in community-dwelling Japanese people aged 40 years and older.^6^ The number of people with diabetes mellitus was relatively small (7.4%) in their study. The presence of diabetes mellitus was not significantly associated with an increased risk of having <20 teeth in participants <50 years and ≥50 years in our study. It is likely that the small number of participants with diabetes mellitus (1.2%) may have influenced the negative result in our study.

### Limitations

There are several limitations in this study. Our study was performed in a uniform group consisting of a large number of Japanese female nurses participating in the JNHS. Although this may be an advantage in some ways in comparison with previous studies, it presents a limitation regarding the generalizability of our findings. The results of our study are not representative of the general population in Japan. This resource-limited environment might have influenced the findings of our study, although the Nurses' Health Study in the United States also evaluated the associations between tooth loss and systemic diseases.^25,26^

The second limitation is the adjustment for the presence of systemic diseases that are associated with periodontal disease progression and/or loss of teeth. Previous cross-sectional studies suggest a significant association between hypertension and tooth loss in postmenopausal women.^27,28^ Alcohol consumption among women was associated with a higher number of teeth present compared with abstainers in the Copenhagen Oral Health Senior Study.^29^ However, no such association was found in the study of Ishikawa et al. in Japan.^6^

The third limitation is the validity of the self-reported number of teeth present. We previously confirmed the association between the actual and self-reported number of teeth present in a different study.^15^ Accordingly, we have applied the self-reported number of teeth present in our other recent studies.^30,31^ Investigators in the Nurses' Health Study in the United States also applied the self-reported number of teeth present in their studies.^25,26^ Ueno et al. demonstrated that the correlation coefficient between clinical and self-reported number of teeth present in 2,356 Japanese adults aged 40–75 years was 0.77.^32^ They concluded that self-reports were considered valid alternatives to clinical measures for estimating number of teeth present.

The fourth limitation is the confounding variables influencing the number of teeth present. Clinical and radiological examinations of oral health status indicators such as dental caries and periodontal disease would give a more comprehensive picture of these factors; however, it is difficult to examine these factors clinically and radiologically in a large cohort study such as the JNHS. Notably, the Nurses' Health Study in the United States did not evaluate oral health status clinically or radiologically.^25,26^ It is likely that inter- and intraobserver reliability among a large number of dentists is relatively low for periodontal examinations charting periodontal pockets, clinical attachment loss, and tooth mobility.

Other socioeconomic status factors associated with the income of participants, such as workplace and family composition, might influence the number of teeth present. Almost all participants worked in a hospital, which indicates that participants' income may be relatively uniform. In addition, most participants were married. This raises the possibility that the family composition of the participants may also be similar in this study.

## Conclusions

Our study showed that Japanese female nurses ≥50 years who had experienced three or more deliveries had a significantly higher risk of having <20 teeth than those ≥50 years who had never delivered. No significant association of parity with the risk of having <20 teeth was observed in female nurses <50 years. Higher-parity female nurses ≥50 years may be more likely to lose teeth than lower-parity female nurses ≥50 years. Higher-parity female nurses ≥50 years should receive regular oral health care in dental clinics to prevent loss of teeth.

## Abbreviations Used

BMI: body mass index
CI: confidence interval
JNHS: Japan Nurses' Health Study
OR: odds ratio
SD: standard deviation
